# Trends and hotspots of energy-based imaging in thoracic disease: a bibliometric analysis

**DOI:** 10.1186/s13244-024-01788-4

**Published:** 2024-08-14

**Authors:** Yufan Chen, Ting Wu, Yangtong Zhu, Jiawei Chen, Chen Gao, Linyu Wu

**Affiliations:** 1 Department of Radiology, The First Affiliated Hospital of Zhejiang Chinese Medical University (Zhejiang Provincial Hospital of Chinese Medicine), Hangzhou, China; 2 The First School of Clinical Medicine, Zhejiang Chinese Medical University, Hangzhou, China

**Keywords:** Bibliometric analysis, X-ray computed tomography, Thoracic disease, Dual-energy X-ray absorptiometry

## Abstract

**Objective:**

To conduct a bibliometric analysis of the prospects and obstacles associated with dual- and multi-energy CT in thoracic disease, emphasizing its current standing, advantages, and areas requiring attention.

**Methods:**

The Web of Science Core Collection was queried for relevant publications in dual- and multi-energy CT and thoracic applications without a limit on publication date or language. The Bibliometrix packages, VOSviewer, and CiteSpace were used for data analysis. Bibliometric techniques utilized were co-authorship analyses, trend topics, thematic map analyses, thematic evolution analyses, source’s production over time, corresponding author’s countries, and a treemap of authors’ keywords.

**Results:**

A total of 1992 publications and 7200 authors from 313 different sources were examined in this study. The first available document was published in November 1982, and the most cited article was cited 1200 times. Siemens AG in Germany emerged as the most prominent author affiliation, with a total of 221 published articles. The most represented scientific journals were the “*European Radiology*” (181 articles, *h*-index = 46), followed by the “*European Journal of Radiology*” (148 articles, *h*-index = 34). Most of the papers were from Germany, the USA, or China. Both the keyword and topic analyses showed the history of dual- and multi-energy CT and the evolution of its application hotspots in the chest.

**Conclusion:**

Our study illustrates the latest advances in dual- and multi-energy CT and its increasingly prominent applications in the chest, especially in lung parenchymal diseases and coronary artery diseases. Photon-counting CT and artificial intelligence will be the emerging hot technologies that continue to develop in the future.

**Critical relevance statement:**

This study aims to provide valuable insights into energy-based imaging in chest disease, validating the clinical application of multi-energy CT together with photon-counting CT and effectively increasing utilization in clinical practice.

**Key Points:**

- Bibliometric analysis is fundamental to understanding the current and future state of dual- and multi-energy CT.
- Research trends and leading topics included coronary artery disease, pulmonary embolism, and radiation dose.
- All analyses indicate a growing interest in the use of energy-based imaging techniques for thoracic applications.

**Graphical Abstract:**

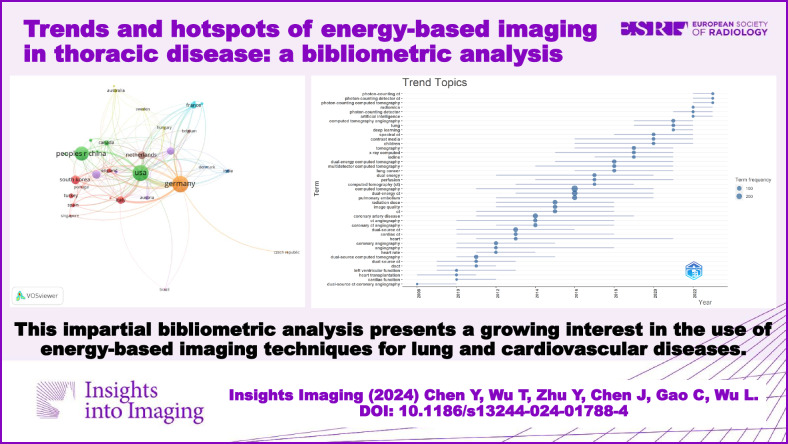

## Introduction

Traditional CT is an important tool for diagnosing thoracic pathologies, including the detection and diagnosis of pulmonary nodules [1], evaluation and follow-up of chronic obstructive pulmonary disease [2], and interstitial lung disease [3]. With advancements in engineering, the diagnostic performance for chest diseases has increased over time. Still, conventional CT images can only provide limited information on the material composition of tissues, such as differentiating between calcified plaques and iodine-containing blood in the vessels [4]. Hence, further advancements are required for a more desirable contrast-to-noise ratio, greater spatial resolution, and reduced radiation dose or energy-based imaging for precise disease diagnosis. In dual-energy CT/multi-energy CT scans, the patients are imaged with two or more X-ray spectra, respectively [5]. Currently, one of the most progressive usages of dual-energy and multi-energy imaging is to form low- and high-keV single-energy images, as well as hybrid images, which allows for better visualization of physiological tissues, improving region of interest area contrast, and reduction of possible metal artifacts [6, 7].

Dual- and multi-energy CT is a potential diagnostic technology for chest diseases with multiple advantages. These technologies can produce high-quality images, enhancing diagnostic accuracy for the early detection of thoracic disease. Furthermore, they have the capability to keep radiation doses at levels as low as reasonably achievable [8]. Because the utilization of dual-energy CT in thoracic disease is rapidly emerging, a study systematically analyzing the current state of this technology can improve clinical practice. Evaluating the research and trends of dual- and multi-energy CT in chest applications will help shed light on the research hotspots and key issues in this field, guiding the direction and focus of future research.

Bibliometric analysis stands as a widely adopted and rigorous methodology for delving into extensive scientific data, unraveling the intricacies of a specific field, and illuminating emerging trends within that domain [9]. By conducting a bibliometric study of dual- and multi-energy CT for chest applications, we can more fully understand and optimize the use of this imaging technique. The objective of this study was to conduct an impartial bibliometric analysis of the trends and hotspots of energy-based imaging in thoracic disease, systematically evaluating the utilization of dual- and multi-energy CT in diagnosing chest disease, as well as to analyze how the scientific interest in this field is growing. Source’s production over time, corresponding author’s countries, treemap of authors’ keywords, trend topics, co-authorship analyses, thematic evolution, as well as thematic map were analyzed to offer a comprehensive review of the prospects and challenges associated with energy CT in thoracic disease. The analysis aimed to spotlight strengths, areas of application, and furnish pertinent references for future research in this field.

## Materials and methods

### Data collection and search strategy

All searches were executed through the Web of Science Core Collection, utilizing its advanced search module. Eligible publications encompassed all pertinent literature, including articles and review articles, up until the search date. The last search was conducted on the 24th of October 2023, without constraints on time or language. The search employed specific strings such as dual-energy CT and (pulmonary or chest or heart or coronary artery). The detailed search strategy is available as supplementary material. All data was extracted from the database on the 24th of October 2023, rendering ethical statements or approval unnecessary.

### Data processing

All data was imported into R software (version 4.3.3) with the Bibliometrix package. Two reviewers independently reviewed the title, abstract, and keywords of each article for inclusion of research content on dual- and multi-energy CT applied to the pulmonary or chest or heart or coronary artery. Articles and review articles with incomplete research information, meeting abstracts, editorial materials, letters, corrections, retrieved publications, book chapters, and duplicate articles were excluded. Publications describing only single-energy CT related applications or the utilization of dual- and multi-energy CT in other areas, such as the abdomen, head, bones, and limbs were also excluded. A specific study flow chart was provided in supplementary materials (Fig. S1).

### Data analysis

For the thematic evolution and mapping, the Bibliometrix was used [10]. The dataset was arbitrarily divided into three segments to analyze thematic evolution. Clustering parameters included author’s keywords (cluster labeling by author’s keywords, number of words set at 250, minimum cluster frequency per thousand documents at 5, labels per cluster at 3, label size at 0.3, and the clustering algorithm used was walk trap). Callon’s centrality and density indices were then computed on the resulting clusters to assess the relevance and degree of development of each theme [11]. Callon’s centrality signifies the theme’s significance within the overall field, while Callon’s density measures the theme’s development [12]. Based on the centrality and density values, the author’s keywords were categorized into four themes: (1) motor (well-developed and highly relevant); (2) basic (highly relevant and underdeveloped); (3) emerging or declining (underdeveloped and low relevance); and (4) niche (well-developed and low relevance). Additionally, the Bibliometrix package was utilized for analyzing the source’s production over time, the corresponding author’s countries, a treemap of the author’s keywords, and trending topics.

For co-authorship analyses of countries, VOSviewer (version 1.6.19) was employed [13]. In the network, each node represents a country, with the size of the node indicating the frequency of occurrences. The links between nodes signify co-occurrences between countries, and the thickness of the link represents the frequency of these co-occurrences. Larger nodes indicate higher occurrences of the country, and thicker links indicate more frequent co-occurrences between countries [14]. For knowledge maps of author co-authorship, CiteSpace (version 6.3.R1) was used [15]. In these knowledge maps, nodes represent authors, the size of the rings around each node corresponds to the number of publications associated with that author, the lines represent the relationship between nodes, and the thickness of these lines between each node indicates the strength of the links between the data [16]. The results from these analyses collectively provide insights into the current research status and trends in the field.

## Results

### Publications and citations

**Fig. 1:**
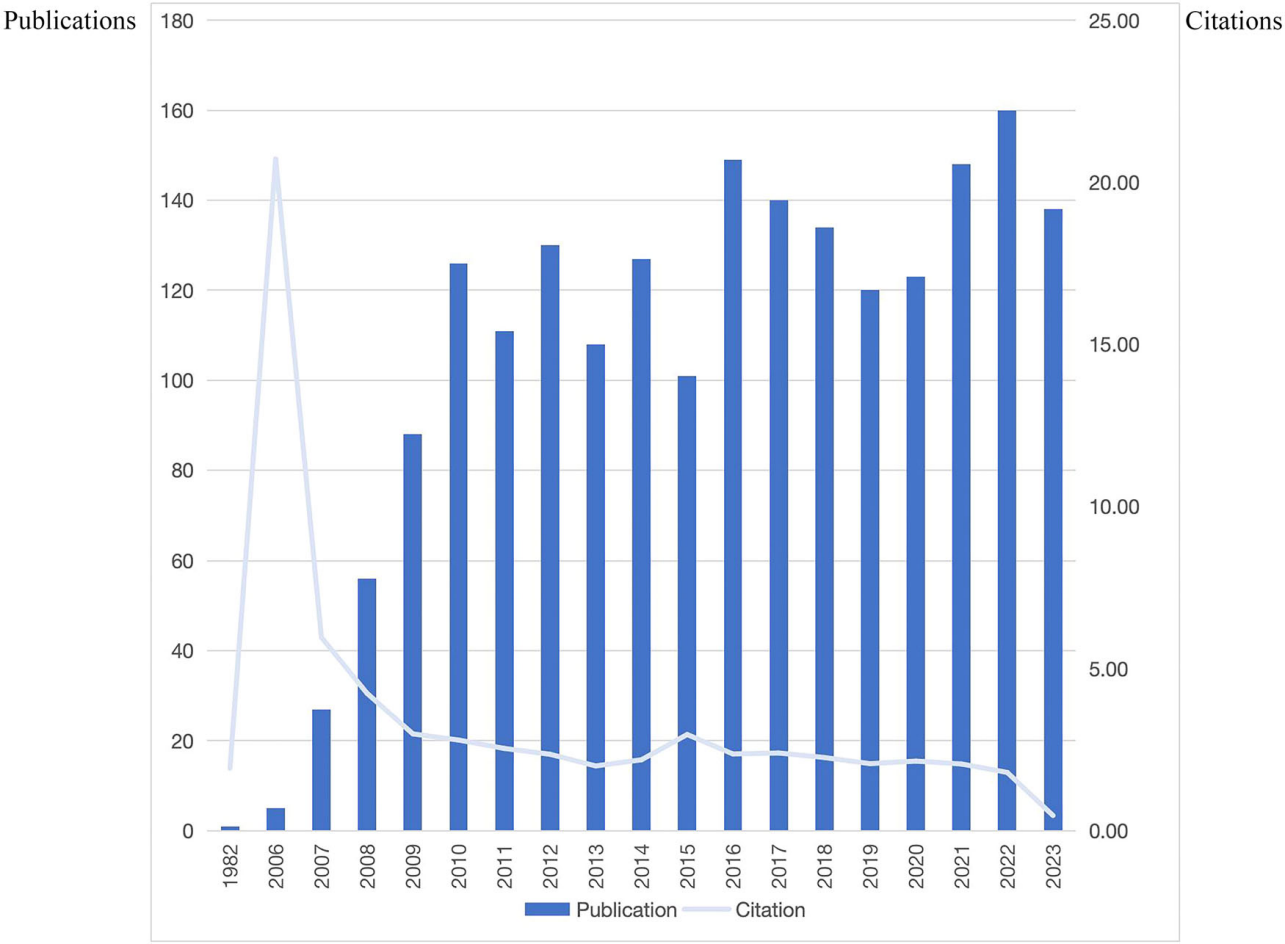
Publications and citations over time

A total of 5906 publications were retrieved, excluding the following: (1) other types besides articles or review articles or proceeding paper or early access (*n* = 282); (2) publications based solely on abdomen or head or bones or limbs and publications based solely on single-energy CT (*n* = 3632), and finally 1992 publications were included in the analysis with 1850 articles (92.9%) and 142 reviews (7.1%). The earliest article was published in November 1982 [17]. The cumulative citations amounted to 66.29, with an average of 3.49 citations per year (Fig. 1). Over the period from 1982 to 2023, the annual publications exhibited fluctuations but demonstrated an overall increasing trend. No literature was published for 23 years after the publication of a related paper in 1982, until the introduction of dual-source CT in 2006, and research in the field is increasingly hot.

Obviously, in 2006, there was a citation peak, with 20.72 articles being cited. The total number of citations in this field declined after a surge in 2006, and gradually declined after a slight increase in 2015. Given that the articles included in the study could not cover the end of 2023, the number of citations in 2023 is low from the graph. As shown in Table 1, the ten most globally cited documents have been identified, and these represent important contributions to this field. The most cited article was cited 1200 times, the second and third most cited articles were cited 1061 and 908 times, respectively, and the rest were cited more than 270 times.

**Table 1:** List of the ten most globally cited documents

Paper	DOI	Total citations	TC per year
JOHNSON TRC, 2007, EUR RADIOL	10.1007/s00330-006-0517-6	1200	66.67
FLOHR TG, 2006, EUR RADIOL	10.1007/s00330-005-2919-2	1061	55.84
MCCOLLOUGH CH, 2015, RADIOLOGY	10.1148/radiol.2015142631	908	90.80
MEIJBOOM WB, 2008, J AM COLL CARDIOL	10.1016/j.jacc.2008.05.024	486	28.59
ACHENBACH S, 2010, EUR HEART J	10.1093/eurheartj/ehp470	465	31.00
CHRISTNER JA, 2010, AM J ROENTGENOL	10.2214/AJR.09.3462	349	23.27
BLANKSTEIN R, 2009, J AM COLL CARDIOL	10.1016/j.jacc.2009.06.014	316	19.75
SCHEFFEL H, 2006, EUR RADIOL	10.1007/s00330-006-0474-0	309	16.26
ACHENBACH S, 2006, EUR J RADIOL	10.1016/j.ejrad.2005.12.017	298	15.68
FEUCHTNER GM, 2009, J AM COLL CARDIOL	10.1016/j.jacc.2008.01.077	271	16.94

*DOI* digital object identifier

### Sources

**Fig. 2:**
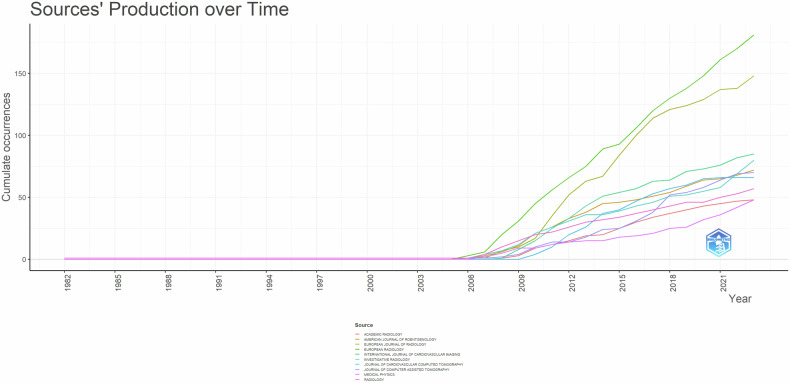
Top ten sources’ production over time

A total of 313 distinct document sources were identified. Among scientific journals, “*European Radiology*” emerged as the most represented source (181 articles, *h*-index = 46), followed by “*European Journal of Radiology*” (148 articles, *h*-index = 34), “*International Journal of Cardiovascular Imaging*” (85 articles, *h*-index = 20). The productivity of the top ten active journals in the past years is shown in Fig. 2, and since 2006 “*European Radiology*” and “*European Journal of Radiology*” gradually led others, offering a rapid development trend.

### Authors and collaborations

Overall, 7200 different authors were included in the analyzed documents. An average number of 7.78 co-authors per document have been esteemed, and 19 single-authored documents were found. The five most productive authors were Schoepf UJ, 119 documents; Alkadhi H, 82 papers; Schmidt B, 64 documents; Achenbach S, 53 documents; De Cecco CN, 52 papers; together with Vogl TJ, 52 papers (Table 2).

**Table 2:** List of top ten authors and cited authors

Rank	Authors	Articles	Cited author	Local citations
1	SCHOEPF UJ	119	SCHOEPF UJ	1357
2	ALKADHI H	82	SCHMIDT B	1205
3	SCHMIDT B	64	ALKADHI H	1180
4	ACHENBACH S	53	FLOHR T	1020
5	DE CECCO CN	52	REISER MF	1018
6	VOGL TJ	52	LESCHKA S	936
7	LESCHKA S	48	ACHENBACH S	920
8	NIKOLAOU K	45	NIKOLAOU K	913
9	FLOHR T	42	JOHNSON TRC	881
10	STOLZMANN P	39	KRAUSS B	819

Siemens AG, Germany, was the most relevant authors’ affiliation based on the number of published articles (*n* = 221), followed by the University Zurich Hospital, Switzerland (*n* = 159), and Harvard University, USA (*n* = 120). The full list of the top ten most relevant authors’ affiliations is represented in Table 3.

**Table 3:** List of the top ten most relevant authors’ affiliations

Affiliation	Articles
SIEMENS AG	221
UNIVERSITY ZURICH HOSPITAL	159
HARVARD UNIVERSITY	120
MEDICAL UNIVERSITY OF SOUTH CAROLINA	115
UNIVERSITY OF ZURICH	101
SIEMENS GERMANY	100
MASSACHUSETTS GENERAL HOSPITAL	93
UNIVERSITE DE LILLE	92
RUPRECHT KARLS UNIVERSITY HEIDELBERG	89
UNIVERSITY OF MUNICH	86

**Fig. 3:**
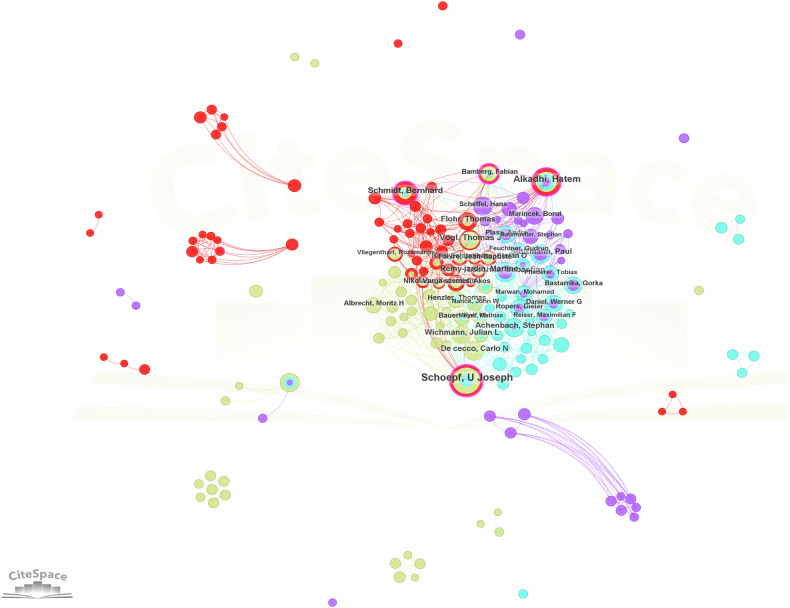
Knowledge maps of author co-authorship. The nodes in the figures represent the authors; the larger the node, the more published articles there are. The lines between the nodes demonstrate the cooperative relationship between them. The colors of the nodes, lines, and cluster outlines are categorized by year

The co-authorship study between authors is depicted in Fig. 3. Notably, the node of Schoepf, U Joseph, stands out as the largest, indicating extensive collaboration with other authors. Schoepf forms an intricate academic cooperation network, closely collaborating with authors such as Alkadhi, Hatem, and others. The top five co-authors were Schoepf, U Joseph (*n* = 111), Alkadhi, Hatem (*n* = 78), Schmidt, Bernhard (*n* = 60), Vogl, Thomas J (*n* = 47), Leschka, Sebastian (*n* = 41).

### Countries and regions

**Fig. 4:**
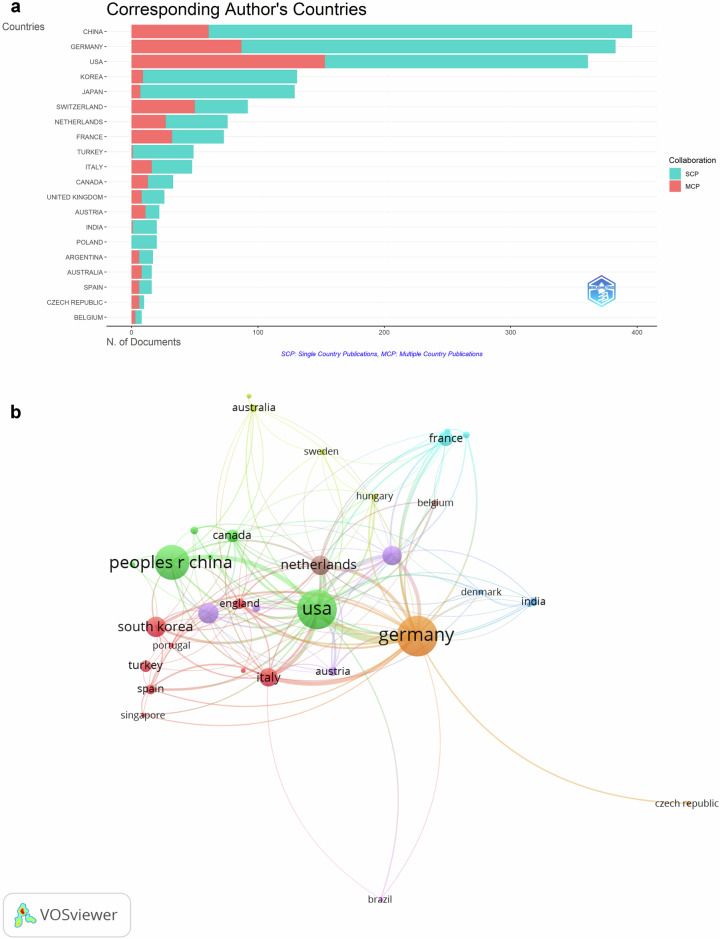
**a** Corresponding author’s countries chart, divided by single country publications (SCP) and multiple country publications (MCP). **b** The network of countries/regions’ co-authorship analysis. Co-authorship analysis of countries with at least five documents. An item’s weight determines the label’s size and the item’s circle, which, in this case, is the number of documents. Lines connecting items represent links. Line thickness represents the power of connections between countries. The visualized distance between the two countries approximates their relatedness. The closer the two countries are related, the closer they are. An item’s color is determined by the cluster to which it belongs

An examination of the corresponding authors’ countries was presented, with a further breakdown into single and multiple country publications (Fig. 4a). China leads with 396 documents published, boasting a single country publication ratio (SCPr) of 84.6%. Following closely were Germany with 383 papers and a SCPr of 77.3%, then the USA with 361 documents and a SCPr of 57.6%.

The co-authorship analysis of countries is visually represented in Fig. 4b. Based on total link strength, the top five countries were the United States (*n* = 496), Germany (*n* = 429), Italy (*n* = 167), Netherlands (*n* = 152), and China (*n* = 118). The USA exhibits extensive cooperative relations with numerous countries, with the highest total link strength. Comparatively, there is less intense cooperation among other countries in the network.

### Keywords and topics

**Fig. 5:**
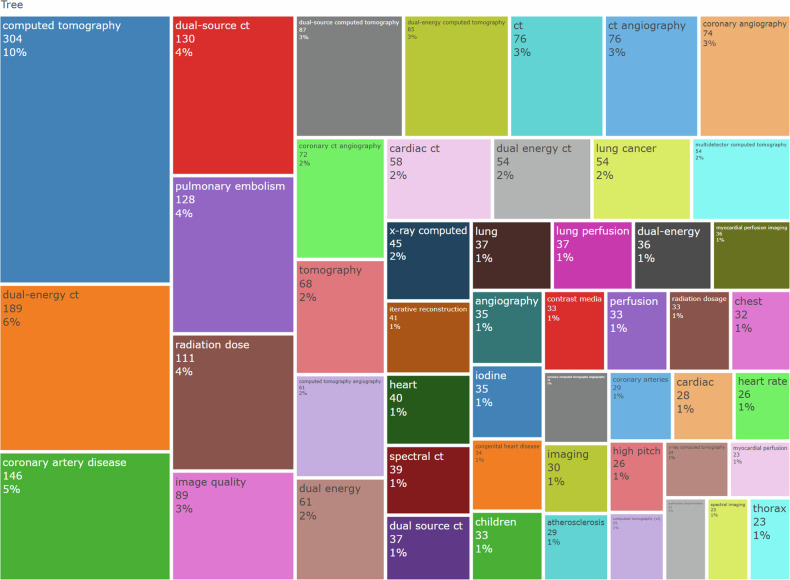
Tree-map of the 50 most frequent author’s keywords

**Fig. 6:**
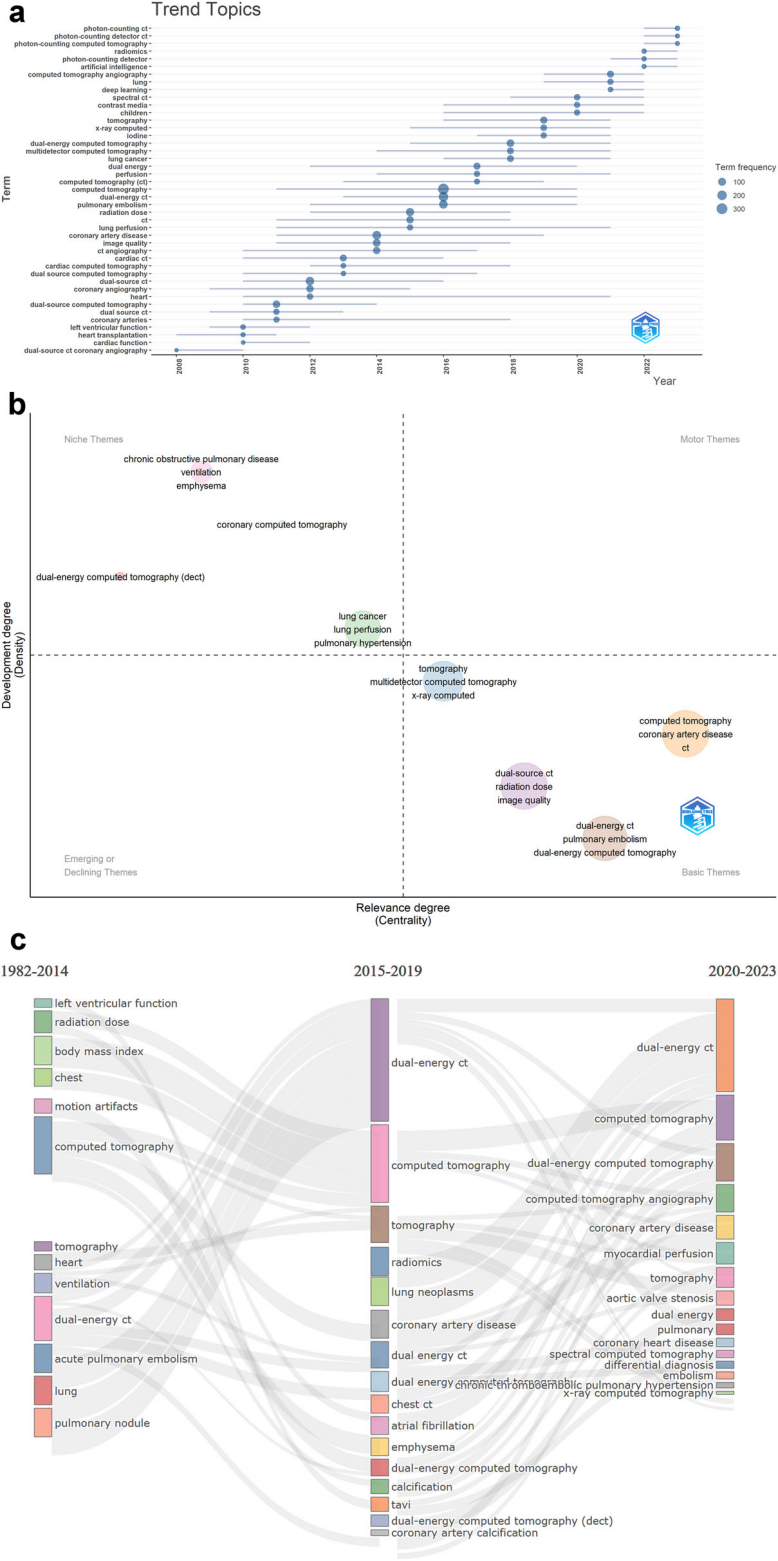
**a** Trend topics of author’s keywords from 2008 to 2023. **b** Thematic evolution of author’s keywords by time. **c** Thematic evolution of author’s keywords. The edges represent the flowing data, the flows represent the specific values of the data flow, the nodes represent the different classifications, and the width of the edges is proportional to the flow: the wider the edge, the larger the value

The author’s keywords of the articles were examined to evaluate the various topics related to the application of dual- and multi-energy CT in the chest. The treemap in Fig. 5 illustrates the 50 most frequent author’s keywords, and the top three keywords were computed tomography (10%), dual-energy CT (6%), and coronary artery disease (5%). The trend topics from 2008 to 2023 have been performed in Fig. 6a, showing the frequency of each author’s keyword at a given time and the period of its continuation. The time span selected when setting the analysis parameters was still 1982-2023. As shown in the figure, emerging hotspots in recent years focus on photon-counting CT, radiomics, artificial intelligence, and deep learning.

The thematic evolution of the author’s keywords over time was depicted in Fig. 6b. Within the “basic themes” cluster were keywords such as tomography, computed tomography, dual-energy CT, and dual-source CT. The cluster containing dual-energy computed tomography, coronary computer tomography, chronic obstructive pulmonary disease, and lung cancer was a “niche theme”. Thematic map analysis based on the four identified clusters is shown in Fig. 6c. This graph indicates each keyword’s node size and evolution in three periods. Among these, dual-energy CT and computed tomography had large nodes with wide edges in all three time periods, and the specific application of these techniques to the chest varied between periods.

## Discussion

In this study, diverse research components have been objectively analyzed to evaluate the current status of research and development trends in the applications of energy-based CT in thoracic disease. Clinically, dual-energy CT exploits two X-rays to collect energy spectra to distinguish the molecules that make up the body tissues according to their atomic number, and it has the ability to combine datasets obtained at various energy levels to achieve substance decomposition, as well as characterization [18–20]. Currently, six approaches exist for dual-energy imaging: single-source sequential, single-source helical, single-source twin-beam, single-source rapid switching, dual-source and dual-layer [21]. In recent years, photon-counting detector CT has technologically enabled true multi-energy CT scanning and has been applied in a number of preclinical and clinical studies [22]. The ultra-high spatial resolution of photon-counting detector CT design permits lower dose scanning for all body regions, and it is especially helpful in identifying significant imaging findings in the thoracic cavity [23]. Therefore, photon-counting detector CT is a promising technique on the verge of being clinically feasible and has the potential to dramatically change the clinical use of CT in the upcoming decades [24].

Using dual-energy CT has enhanced diagnostic confidence without being exposed to substantial radiation effects, expanding the potential of thoracic imaging in children and adults [25, 26]. Several dual-energy CT platforms can be used to generate or detect two-photon spectra of high energy and low energy, among which Canon Medical System’s two-photon spectra are obtained through different spectral acquisition techniques; GE Healthcare enables ultra-fast kvp switching, including fast switching from 80 kVp to 140 kVp, providing nearly simultaneous spatial and temporal registration of projections; Philips Medical chose to use double-layer detector technology (i.e., nano-panel prism detector), which can obtain two-photon spectra simultaneously; and for Siemens Healthineers, the two-photon spectra are acquired in the acquisition process of two different dual-energy CT platforms [27]. With the swift development of technology, interest in energy-based imaging-related applications is increasing, and radiologists have started investigating thoracic disorders in unprecedented conditions, such as analysis of bronchopulmonary disorders and cardiovascular diseases.

To the best of our knowledge, there are general or systematic reviews of dual- and multi-energy CT in thoracic disease [28–30] but no more well-developed bibliometric studies. Our study provides insights into the specifics of publications for each of the countries under consideration. Concerning international groups, our data reveal that the number of multiple country publications varies among the top ten countries, suggesting that collaboration is not yet widespread across countries. China, Germany, and the United States are the top three countries in terms of the number of publications, but China’s articles are cited less than the other two countries at the top of the ranking, indicating that there is still a certain gap between developing and developed countries. Further analysis of the countries of co-authorship with at least five publications was performed. There are nine clusters of different sizes, and the largest of these clusters is cluster 2, which includes the USA, China, Canada, and so on, showing that cooperation is still mostly between countries with better economic development. These countries may be more advantaged in terms of investment and development of medical resources and have been able to introduce and apply advanced medical imaging technologies, including dual-energy CT, earlier. As a result, these countries have more opportunities and capacity to conduct research in the medical field and publish their results as academic papers. The United States occupies a key position in the co-author collaboration network, indicating its relatively leading position in dual- and multi-energy CT.

An analysis of the top ten most referenced documents has been conducted to discern the significance of articles that have had a crucial impact on the scientific literature in the field we studied. These ten articles concentrate on the principles, dosage, and quality of dual-energy CT and its chest applications. The top three most cited articles were the most representative and made the greatest contribution, which deserves further in-depth analysis. The first most cited article was written in 2007 [31] by Johnson TR et al and the second most referenced literature was authored by Flohr et al in 2006 [32]; both papers demonstrate that dual-energy CT enjoys a large potential and quite a lot of conceivable clinical advantages in CT angiography. The purpose of the former was to evaluate the feasibility of using dual-energy CT to distinguish iodine from other materials and of different body tissues, while the latter demonstrated an interesting application of dual-energy CT to separate bones and iodine-filled blood vessels in CT angiography. The third most referenced article, authored by McCollough et al in 2015 [33] systematically introduces the underlying motivation and physical principles of dual- and multi-energy CT. Also, the current technical approaches and evolving clinical applications are described, laying a favorable foundation for subsequent related research. It demonstrates that dual- and multi-energy CT is steadily becoming mainstream CT imaging and has a promising future. As for scientific journals, the top two journals by *h*-index are *European Radiology* (*h*-index = 46) and *Radiology* (*h*-index = 36), while *European Radiology* emerges as the most represented source, reflecting its academic standing and high influence in the field of energy-based imaging. Of the top three most cited articles, the first and second are from *European Radiology*, and the third is from *Radiology*, so it is expected that high-quality articles with major breakthroughs are published in such excellent journals.

Trending topics showed the changes of each author’s keywords according to the timeline, which meant that the issues of dual- and multi-energy CT have changed over time. The related topics in 2008 were few and low-frequency, representing that dual- and multi-energy CT was in the preliminary application stage back then. By 2016, the application of dual-energy CT became a relatively common trend. Our analysis reveals that dual- and multi-energy CT is increasingly being applied to lungs, blood vessels, and heart. The results of the trending topics analysis show that lung-related keywords also appeared with higher frequency recently, indicating that dual-energy CT was gradually applied to the lung. In terms of lung applications, dual-energy CT can provide anatomical and functional information about the lung in diverse pulmonary disease states which can effectively improve the diagnosis of acute and chronic pulmonary embolisms, pulmonary malignancies, and lung parenchymal diseases, as well as open up new avenues for lung imaging [34]. Spectral photon-counting CT can achieve higher spatial resolution, which not only allows for earlier detection and more precise classification of pulmonary nodules, but also improves diagnostic confidence for radiologists to evaluate other lung abnormalities, such as airway and parenchymal diseases [35, 36]. The results of the thematic map analysis show that the most important dual- and multi-energy CT applications in 2020–2023 include myocardial perfusion, coronary artery disease, aortic valve stenosis, and chronic thromboembolic pulmonary hypertension. In vascular applications, dual-energy CT is supposed to break through the limitations of standard monoenergetic CT angiography, including patient exposure to carcinogenic radiation and nephrotoxic contrast agents, inadequate contrast vascular enhancement, interference from metal and beam hardening artifacts, and serious vascular calcification, as well as limited tissue characterization together with perfusion evaluation [37]. In terms of cardiac imaging evaluation, several applications of cardiac dual-energy CT, such as virtual non-contrast reconstruction, virtual single-energy images, as well as iodine myocardial perfusion diagrams, have been shown to improve both diagnostic accuracy and image quality simultaneously reduce radiation and contrast agent administration [38].

Admittedly, there are still some limitations to this analysis. Firstly, as the literature search was conducted on the 26th of October 2023, we could not cover all publications and citations in 2023, which would have led to the omission of current hot topics. Secondly, only a single database (the Web of Science Core Collection) was retrieved in this study, making the results relatively biased. In the future, other databases such as PubMed and Scopus can be jointly used to obtain more objective and comprehensive results. Additionally, the possibilities for designing a search strategy are diverse, resulting in some publications not being considered and a bias in the number of citations, which may at least partially affect the results of our findings.

The bibliometric examination offers a comprehensive summary of the current status and benefits of dual- and multi-energy CT, paving the way for future clinical diagnostic and analytical applications in thoracic disease. Notably, dual- and multi-energy CT has witnessed rapid development in recent years, and its advantageous application in lung parenchymal diseases and coronary artery diseases has become increasingly prominent, indicating a growing utilization in chest diseases with a promising trajectory for future development. In the future, photon-counting CT and artificial intelligence will be hot technologies that continue to develop. In essence, the evolution of dual- and multi-energy CT is anticipated to drive innovations across various domains.

### Supplementary information

ELECTRONIC SUPPLEMENTARY MATERIAL

## Data Availability

All data generated or analyzed during this study are included in this published article and its additional files.

## Abbreviation

SCPr: Single country publication ratio

## References

[1] Liang X, Kong Y, Shang H et al (2022) Computed tomography findings, associated factors, and management of pulmonary nodules in 54,326 healthy individuals. J Cancer Res Ther 18:2041–2048 36647968 10.4103/jcrt.jcrt_1586_22

[2] Bakker JT, Klooster K, Vliegenthart R, Slebos DJ (2021) Measuring pulmonary function in COPD using quantitative chest computed tomography analysis. Eur Respir Rev 30:210031 34261743 10.1183/16000617.0031-2021 PMC9518001

[3] Iwasawa T, Matsushita S, Hirayama M, Baba T, Ogura T (2023) Quantitative analysis for lung disease on thin-section CT. Diagnostics 13:2988 37761355 10.3390/diagnostics13182988 PMC10528918

[4] Patino M, Prochowski A, Agrawal MD et al (2016) Material separation using dual-energy CT: current and emerging applications. Radiographics 36:1087–1105 27399237 10.1148/rg.2016150220

[5] Yang M, Wohlfahrt P, Shen C, Bouchard H (2023) Dual- and multi-energy CT for particle stopping-power estimation: current state, challenges and potential. Phys Med Biol 68:04TR1. 10.1088/1361-6560/acabfa 10.1088/1361-6560/acabfa 36595276

[6] Rassouli N, Etesami M, Dhanantwari A, Rajiah P (2017) Detector-based spectral CT with a novel dual-layer technology: principles and applications. Insights Imaging 8:589–598 28986761 10.1007/s13244-017-0571-4 PMC5707218

[7] Adam SZ, Rabinowich A, Kessner R, Blachar A (2021) Spectral CT of the abdomen: Where are we now? Insights Imaging 12:138 34580788 10.1186/s13244-021-01082-7 PMC8476679

[8] Godreau JP, Vulasala SSR, Gopireddy D (2022) Introducing and building a dual-energy CT business. Semin Ultrasound CT MR 43:355–363 35738821 10.1053/j.sult.2022.03.005

[9] Donthu N, Kumar S, Mukherjee D, Pandey N, Lim WM (2021) How to conduct a bibliometric analysis: an overview and guidelines. J Bus Res 133:285–296 10.1016/j.jbusres.2021.04.070

[10] Aria M, Cuccurullo C (2017) bibliometrix: an R-tool for comprehensive science mapping analysis. J Informetr 11:959–975 10.1016/j.joi.2017.08.007

[11] Aria M, Misuraca M, Spano M (2020) Mapping the evolution of social research and data science on 30 years of social indicators research. Soc Indic Res 149:803–831 10.1007/s11205-020-02281-3

[12] Kocak B, Baessler B, Cuocolo R, Mercaldo N, Pinto Dos Santos D (2023) Trends and statistics of artificial intelligence and radiomics research in radiology, nuclear medicine, and medical imaging: bibliometric analysis. Eur Radiol 33:7542–7555 37314469 10.1007/s00330-023-09772-0

[13] van Eck NJ, Waltman L (2010) Software survey: VOSviewer, a computer program for bibliometric mapping. Scientometrics 84:523–538 20585380 10.1007/s11192-009-0146-3 PMC2883932

[14] Qi M, Ren JM (2023) An overview and visual analysis of research on government regulation in healthcare. Front Public Health 11:1272572 38026398 10.3389/fpubh.2023.1272572 PMC10679357

[15] Chen C (2006) CiteSpace II: detecting and visualizing emerging trends and transient patterns in scientific literature. J Am Soc Inform Sci Technol 57:359–377 10.1002/asi.20317

[16] Wu Y, Wang H, Wang Z, Zhang B, Meyer BC (2019) Knowledge mapping analysis of rural landscape using CiteSpace. Sustainability 12:1–17 10.3390/su12010066

[17] Cann CE, Gamsu G, Birnberg FA, Webb WR (1982) Quantification of calcium in solitary pulmonary nodules using single- and dual-energy CT. Radiology 145:493–496 7134457 10.1148/radiology.145.2.7134457

[18] Forghani R, De Man B, Gupta R (2017) Dual-energy computed tomography: physical principles, approaches to scanning, usage, and implementation: part 1. Neuroimaging Clin N Am 27:371–384 28711199 10.1016/j.nic.2017.03.002

[19] Forghani R, Srinivasan A, Forghani B (2017) Advanced tissue characterization and texture analysis using dual-energy computed tomography: horizons and emerging applications. Neuroimaging Clin N Am 27:533–546 28711211 10.1016/j.nic.2017.04.007

[20] Parakh A, Macri F, Sahani D (2018) Dual-energy computed tomography: dose reduction, series reduction, and contrast load reduction in dual-energy computed tomography. Radiol Clin North Am 56:601–624 29936950 10.1016/j.rcl.2018.03.002

[21] Cicero G, Ascenti G, Albrecht MH et al (2020) Extra-abdominal dual-energy CT applications: a comprehensive overview. Radiol Med 125:384–397 31925704 10.1007/s11547-019-01126-5

[22] Jacobsen MC, Thrower SL, Ger RB et al (2020) Multi-energy computed tomography and material quantification: Current barriers and opportunities for advancement. Med Phys 47:3752–3771 32453879 10.1002/mp.14241 PMC8495770

[23] Esquivel A, Ferrero A, Mileto A et al (2022) Photon-counting detector CT: key points radiologists should know. Korean J Radiol 23:854–865 36047540 10.3348/kjr.2022.0377 PMC9434736

[24] Willemink MJ, Persson M, Pourmorteza A, Pelc N, Fleischmann D (2018) Photon-counting CT: technical principles and clinical prospects. Radiology 289:293–312 30179101 10.1148/radiol.2018172656

[25] Rapp JB, Biko DM, Siegel MJ (2023) Dual-energy CT for pediatric thoracic imaging: a review. AJR Am J Roentgenol 221:526–538 37195790 10.2214/AJR.23.29244

[26] Otrakji A, Digumarthy SR, Lo Gullo R, Flores EJ, Shepard JA, Kalra MK (2016) Dual-energy CT: spectrum of thoracic abnormalities. Radiographics 36:38–52 26761530 10.1148/rg.2016150081

[27] Greffier J, Villani N, Defez D, Dabli D, Si-Mohamed S (2023) Spectral CT imaging: technical principles of dual-energy CT and multi-energy photon-counting CT. Diagn Interv Imaging 104:167–177 36414506 10.1016/j.diii.2022.11.003

[28] Rapp JB, Biko DM, White AM, Ramirez-Suarez KI, Otero HJ (2022) Spectral imaging in the pediatric chest: past, present and future. Pediatr Radiol 52:1910–1920 35726069 10.1007/s00247-022-05404-9

[29] Siegel MJ, Ramirez-Giraldo JC (2019) Dual-energy CT in children: imaging algorithms and clinical applications. Radiology 291:286–297 30912717 10.1148/radiol.2019182289

[30] Vulasala SSR, Wynn GC, Hernandez M et al (2022) Dual-energy imaging of the chest. Semin Ultrasound CT MR 43:311–319 35738816 10.1053/j.sult.2022.03.007

[31] Johnson TR, Krauss B, Sedlmair M et al (2007) Material differentiation by dual energy CT: initial experience. Eur Radiol 17:1510–1517 17151859 10.1007/s00330-006-0517-6

[32] Flohr TG, McCollough CH, Bruder H et al (2006) First performance evaluation of a dual-source CT (DSCT) system. Eur Radiol 16:256–268 16341833 10.1007/s00330-005-2919-2

[33] McCollough CH, Leng S, Yu L, Fletcher JG (2015) Dual- and multi-energy CT: principles, technical approaches, and clinical applications. Radiology 276:637–653 26302388 10.1148/radiol.2015142631 PMC4557396

[34] Lu GM, Zhao Y, Zhang LJ, Schoepf UJ (2012) Dual-energy CT of the lung. AJR Am J Roentgenol 199:S40–S53 23097167 10.2214/AJR.12.9112

[35] Kopp FK, Daerr H, Si-Mohamed S et al (2018) Evaluation of a preclinical photon-counting CT prototype for pulmonary imaging. Sci Rep 8:17386 30478300 10.1038/s41598-018-35888-1 PMC6255779

[36] Fletcher JG, Inoue A, Bratt A et al (2024) Photon-counting CT in thoracic imaging: early clinical evidence and incorporation into clinical practice. Radiology 310:e231986 38501953 10.1148/radiol.231986

[37] Machida H, Tanaka I, Fukui R et al (2016) Dual-energy spectral CT: various clinical vascular applications. Radiographics 36:1215–1232 27399244 10.1148/rg.2016150185

[38] Dell’Aversana S, Ascione R, De Giorgi M et al (2022) Dual-energy CT of the heart: a review. J Imaging 8:236 36135402 10.3390/jimaging8090236 PMC9503750

