# Supplementary material for: Trends and hotspots of energy-based imaging in thoracic disease: a bibliometric analysis
Source: Insights Imaging. 2024 Aug 14;15:209. doi: 10.1186/s13244-024-01788-4 (PMC11324624; doi:10.1186/s13244-024-01788-4)

Trends and hotspots of energy-based imaging in thoracic disease: a bibliometric analysis

ELECTRONIC SUPPLEMENTARY MATERIAL

Supplementary Text S1: Syntax details.

Please note all the searches were performed in the advanced search module. In the standard search module, it may result in a syntax error.

#1 881,227
TS=(tomography, x-ray computed) OR TS=(comput* tomography) OR TS=(CT) OR TS=(CAT) OR TS=(MSCT) OR TS=(MDCT) OR TS=(multislice) OR TS=(multidetector) OR TS=(comput* tomographic angiography)

#2 1,983,953
TS=(Dual energy) OR TS=(Dual-energy) OR TS=(Multi energy) OR TS=(Multi-energy) OR TS=(Energy resolved) OR TS=(Energy based) OR TS=(Tube potential switching) OR TS=(Multiple tube potentials) OR TS=(Multi*kV) OR TS=(Multi*kVp) OR TS=(Kv*switching) OR TS=(Kvp*switching) OR TS=(rapid kilovoltage switching) OR TS=(fast kilovoltage switching) OR TS=(Spectral) OR TS=(Iodine Overlay) OR TS=(Iodine map*) OR TS=(Photon counting) OR TS=(Photon-counting) OR TS=(Photon-counting detector) OR TS=(dual source) OR TS=(iodine quantification iodine map) OR TS=(virtual monoenergetic) OR TS=(virtual monochromatic) OR TS=(virtual noncontrast) OR TS=(virtual unenhanced) OR TS=(z effective) OR TS=(Zeff) OR TS=(material decomposition)

#3 3,034,698
TS=(pulmonary) OR TS=(lung) OR TS=(lungs) OR TS=(bronchi*) OR TS=(alveolar) OR TS=(subsolid nodule*) OR TS=(solid nodule*) OR TS=(ground glass nodule*) OR TS=(part solid nodule*) OR TS=(non solid nodule*) OR TS=(partly solid nodule*) OR TS=(thoracic) OR TS=(thorax) OR TS=(chest) OR TS=(mediastina*) OR TS=(pleura*) OR TS=(thymus) OR TS=(thymic) OR TS=(thymoma) OR TS=(trachea*) OR TS=(rib) OR TS=(costa) OR TS=(sternum) OR TS=(heart) OR TS=(coronary artery) OR TS=(thoracic aorta)

#4 5,906
#1 AND #2 AND #3

#5 5,549
#1 AND #2 AND #3 and Article or Review Article (Document Types)

#6 5,626
#1 AND #2 AND #3 and Article or Review Article or Proceeding Paper or Early Access(Document Types)

Supplementary Figure S1: Flow diagram of this study.

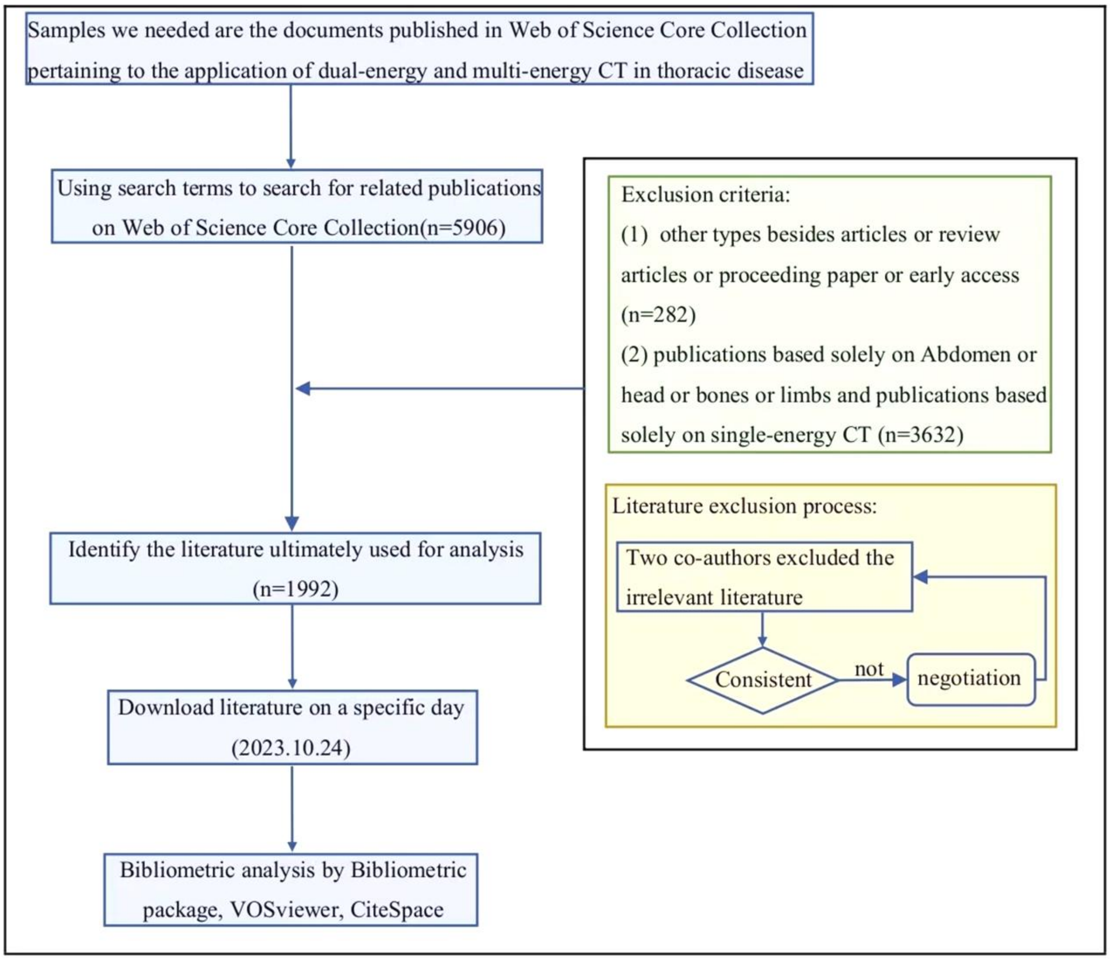

Supplement: Supplementary file 1 — ELECTRONIC SUPPLEMENTARY MATERIAL [file 13244_2024_1788_MOESM1_ESM.pdf]
